# Seroconversion in Wild Birds and Local Circulation of West Nile Virus, Spain

**DOI:** 10.3201/eid1312.070343

**Published:** 2007-12

**Authors:** Jordi Figuerola, Ramon Soriguer, Gema Rojo, Concepción Gómez Tejedor, Miguel Angel Jimenez-Clavero

**Affiliations:** * Consejo Superior de Investigaciones Científicas, Seville, Spain; † Laboratorio Central de Veterinaria, Algete, Madrid, Spain; ‡ Instituto Nacional de Investigación y Tecnología Agraria y Alimentaria, Valdeolmos, Madrid, Spain

**Keywords:** Dispersal, reservoir ecology, seroconversion, seroprevalence, virus circulation, West Nile virus ecology, Spain, dispatch

## Abstract

A serosurvey for neutralizing antibodies against West Nile virus (WNV) in common coots (*Fulica atra*) was conducted in Doñana, Spain. Antibody prevalence was highest in 2003, intermediate in 2004, and lowest in 2005. Some birds seroreverted <1 year after first capture. Seroconversion of birds suggests local circulation of the virus.

In western Mediterranean countries, the frequency of outbreaks of West Nile virus (WNV) infection has increased in recent decades. Evidence for WNV circulation in Spain has remained elusive, although WNV foci have recently been identified in 3 neighboring countries (Morocco, Portugal, and France) (*1*–*3*). Recent WNV activity in Spain has been shown by serologic screening in humans, with detection of WNV-specific immunoglobulin M (*4*) and identification of the first clinical case in 2004 (*5*). In avian hosts, WNV-neutralizing antibodies have been found in chicks of wild migratory birds in southern Spain (*6*). However, interpretation of serologic data is not straightforward because antibodies in chicks may be the result of maternal transmission through eggs (*7*). To ascertain local circulation of WNV in Spain, we designed a capture-recapture study in which serum samples from wild birds were obtained at different times.

## The Study

We focused on the partially migratory common coot (*Fulica atra*) because of its high seroprevalence for WNV detected during a preliminary screening of 72 bird species (J. Figuerola et al., unpub. data). Reasons for this high seroprevalence remain unclear, although preference of this bird for mosquito-rich habitats and its relative size (weight ≈800 g) might be involved in this pattern. Birds were captured in Doñana (37°6′N, 6°9′W) in a walk-in trap in October 2003 (3 capture sessions) and from September through February in 2004–2005 (12 sessions) and 2005–2006 (14 sessions). Overall, 853 captures of 515 different birds were conducted (1–7 captures/bird).

Blood was obtained from the tarsal vein and allowed to clot, and serum was stored at –20°C. All birds were marked with numbered metal rings. Age was determined by plumage characteristics before the birds were released. Neutralizing antibody titers for WNV (strain Eg101) were determined by using a micro-virus neutralization test as described (*6*). Only birds that showed neutralization (absence of a cytopathic effect) at dilutions >1:20 were considered seropositive. Controls for cytotoxicity in the absence of virus were included for every sample at a 1:10 dilution. Cytotoxic samples were excluded from the analysis.

Seroconversion was defined as a bird that was seronegative when first captured and became seropositive at recapture with an antibody titer that had increased 4-fold (*8*). Seroreversion was defined as a seropositive bird whose antibody titer decreased below the cut-off value of 20 at recapture. The interassay coefficient of variation of titers, expressed as log_10_ (calculated using an internal control repeated in 5 different assays, mean 2.56, standard deviation 0.35) was 13.67%. This variation is similar to that observed in individual samples and repeated in different assays. In a series of 27 samples tested twice, the mean fluctuation observed was 0.29 log_10_ units (≈2-fold). To obtain accurate measurements of titers, particularly when assessing seroconversion/seroreversion, we analyzed samples at least twice, and when results differed, they were assayed again until a consistent result was obtained. Specificity of the test was assessed by parallel neutralization against Usutu virus (strain SAAR 1776), a flavivirus found in wild birds that belongs to the same serogroup as WNV, with a panel of sera positive for WNV by micro-virus neutralization test. All titers were higher for WNV than for Usutu virus; 93.6% were >4× higher (Table 1). These results suggested that the neutralizing antibody response was generated by WNV or an antigenically related WNV-like virus.

**Table 1:** Antibody titers against West Nile virus (WNV) and Usutu virus in 47 serum samples from common coots, Doñana, Spain

WNV titer	Usutu virus titer					
	Negative	20	40	80	160	320
20	11	–	–	–	–	–
40	12	2	1	–	–	–
80	10	3	–	–	–	–
160	2	1	1	–	–	–
320	2	2	–	–	–	–

Comparisons between years were restricted to data from October, the only month sampled in all 3 years. For analysis of variation in antibody prevalence within seasons, data were grouped into 2-month intervals. Prevalence was analyzed by generalized linear models with binomial distributed error, logit link, and randomly choosing 1 observation per bird.

**Figure 1:**
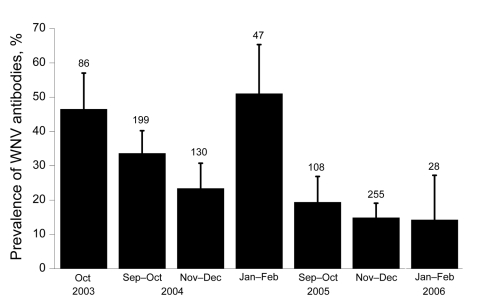
Prevalence of common coots with neutralizing antibodies against West Nile virus (WNV), Doñana, Spain, 2003–2006. Numbers above bars indicate sample size for each period. Error bars show 95% confidence intervals.

Prevalence of WNV-neutralizing antibodies was highest in October 2003, intermediate in October 2004, and lowest in October 2005 (χ^2^ 22.80, df 2, p<0.0001, p<0.05 for all pairwise comparisons) (Figure 1). Juvenile (<1 year of age) birds had lower antibody prevalences than adults in October (χ^2^ 7.14, df 1, p = 0.008). Antibody prevalence increased throughout the 2004–2005 season (χ^2^ 8.45, df 2, p = 0.02), but not during the 2005–2006 season (χ^2^ 1.10, df 2, p = 0.58) (Figure 1).

Of 95 birds captured in 2 consecutive years, 59% had no detectable antibodies in either year, 21% seroreverted, 6.3% seroconverted, and 13.7% had antibodies in both years. Seroconversion confirms that WNV circulation is present in the study area, and seroreversion indicates that antibody titers decreased. Antibodies persisted for >1 year in some birds, although whether this was caused by reinfection, which would stimulate the antibody response, is uncertain.

**Figure 2:**
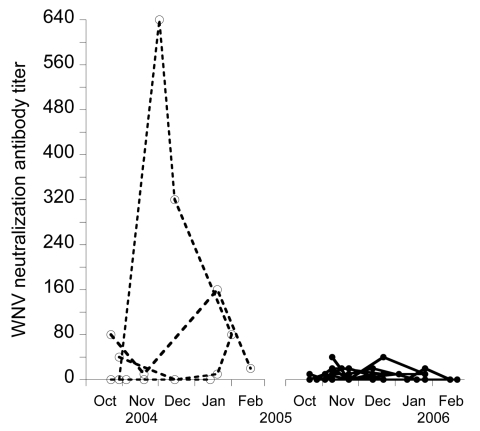
Evolution of West Nile virus (WNV) antibody titers in common coots captured on >4 occasions in the same winter, Doñana, Spain. Open circles and dashed lines indicate birds captured during 2004–2005, and solid circles and continuous lines indicate birds captured during 2005–2006.

Of 54 birds captured at least twice in 2004–2005, 16.7% seroconverted (Table 2), 3.7% seroreverted, 46.2% never had any detectable antibodies, and 33.3% had antibodies whenever captured. This high rate of seroconversion, together with the few seroreversions observed, resulted in high seroprevalence, which reflects high WNV activity during this period. In 2005–2006, of 114 birds, 8.8% seroconverted, 15.8% seroreverted, 65.8% never had any detectable antibodies, and 2.6% had antibodies whenever captured. Antibody prevalence decreased in 2005–2006 (Figure 1), and antibody titers decreased to values near the cut-off point (Figure 2), which made changes in antibody status difficult to interpret. However, the most likely reason for these changes were fluctuations in titers (from undetectable to 10 to 20) (Figure 2) because 7% of the birds showed changes in titers (from 10 to 20) at recapture.

**Table 2:** Seroconversion results for antibodies to West Nile virus in 9 common coots, Doñana, Spain, 2004–2005

Bird ring no.	Age	Date of capture before seroconversion	Date of recapture (antibody titer)	No. days between captures
7060424	Juvenile	2004 Sep 30	2005 Feb 17 (40)	130
7060486	Adult	2004 Dec 2	2005 Feb 1 (640)	51
7069114	Juvenile	2004 Nov 19	2005 Feb 17 (80)	80
7069137	Juvenile	2004 Nov 19	2005 Jan 20 (20)	52
7069177	Adult	2004 Dec 15	2005 Jan 20 (160)	37
7073621	Juvenile	2004 Dec 2	2004 Dec 15 (640)	14
7073622	Juvenile	2004 Oct 29	2004 Dec 2 (640)	35
7073647	Juvenile	2004 Nov 4	2005 Feb 17 (40)	95
7081027	Adult	2004 Dec 15	2005 Jan 20 (320)	37

## Conclusions

We provide evidence for local circulation of WNV in our study area during 2004–2005. This evidence was obtained just a few months after a reported outbreak of WNV that affected humans in Algarve, Portugal, ≈100 km west of our study area. However, no increase in clinical signs or mortality rates was observed in the common coot population during the study period. The high prevalence of antibodies in juvenile birds in September–October 2003 (37.5%) and 2004 (28.8%) also suggests that WNV may have been circulating during summer and autumn of 2003 and 2004.

WNV circulation decreased to low levels or was absent during the 2005–2006 winter season. There are several nonexclusive explanations for this pattern. First, the virus may not easily overwinter in Spain and thus needs to be reseeded each spring by migratory birds arriving from Africa. Nevertheless, climatic conditions probably enable the virus to survive winter because mosquitoes are present year round in the area (Servicio de Control de Mosquitos, unpub. data), and seroconversion in common coots occurred by midwinter. Second, in 2005, a severe drought reduced habitat for both mosquitoes and waterbirds. Third, high seroprevalence at the end of the winter of 2005 would have resulted in effective herd immunity, which may have reduced the number of available hosts in 2006 and transmission intensity. Although this negative loop is only valid if the rate of host population turnover is low (*9*), the scarcity of immunologically naive juvenile birds during 2005–2006 makes this a reliable alternative.

Additional studies are needed to evaluate the role of these 3 mechanisms in the dynamics of WNV in Spain. Combining serologic results for common coots and vector sampling for virus detection may provide information needed to address these issues.
